# Fine-Tuning of Type I Interferon Response by STAT3

**DOI:** 10.3389/fimmu.2019.01448

**Published:** 2019-06-26

**Authors:** Ming-Hsun Tsai, Li-Mei Pai, Chien-Kuo Lee

**Affiliations:** ^1^Graduate Institute of Immunology, National Taiwan University College of Medicine, Taipei, Taiwan; ^2^Department of Biochemistry and Molecular Biology, Chang Gung University, Taoyuan, Taiwan; ^3^Molecular Medicine Research Center, Chang Gung University, Taoyuan, Taiwan; ^4^Liver Research Center, Chang Gung Memorial Hospital, Taoyuan, Taiwan

**Keywords:** type I interferon (IFN-I), STAT3, phospholipid scramblase 2, antiviral immunity, SOCS3

## Abstract

Type I interferon (IFN-I) is induced during innate immune response and is required for initiating antiviral activity, growth inhibition, and immunomodulation. STAT1, STAT2, and STAT3 are activated in response to IFN-I stimulation. STAT1, STAT2, and IRF9 form ISGF3 complex which transactivates downstream IFN-stimulated genes and mediates antiviral response. However, the role of STAT3 remains to be characterized. Here, we review the multiple actions of STAT3 on suppressing IFN-I responses, including blocking IFN-I signaling, downregulating the expression of ISGF3 components, and antagonizing the transcriptional activity of ISGF3. Finally, we discuss the evolution of the suppressive activity of STAT3 and the therapeutic potential of STAT3 inhibitors in host defense against viral infections and IFN-I-associated diseases.

## STAT3, an Overlooked Signal Mediator of IFN-I

STAT3 was originally identified as acute-phase response factor (APRF) that is activated by IL-6 and binds to the promoters of acute-phase protein genes in hepatocytes to regulate inflammatory responses (1–4). It is now known that STAT3 is widely expressed in different cells and is activated by an array of cytokines and growth factors to mediate various activities (5–8). Total body ablation of STAT3 results in embryonic lethality (9, 10), whereas tissue-specific knockout of STAT3 reveals multiple functions in immune system, including regulation of homeostasis of immune cells, such as B-cells (11) and granulocytes (12), survival and/or proliferation of thymocytes (13), and differentiation of plasma cells (14), Th2 (15), Treg (16), and follicular helper T (Tfh) cells (17). Although STAT3 controls RORγt expression and Th17 development (18, 19), it, however, is not required for generation of type 3 innate lymphoid cells (ILC3) whose master regulator is also RORγt (20).

Like other STAT proteins, the activity of STAT3 is also regulated by acetylation, methylation and other post-translational modification such as SUMOylation in addition to phosphorylation (21, 22). STAT3 is activated primarily by tyrosine phosphorylation at Y705. In addition, serine phosphorylation at S727 is required for full transactivation ability of STAT3. However, unphosphorylated STAT3 can still form dimers through its N-terminal domain (NTD) and induce transcription (23). Moreover, acetylation of STAT3 plays a positive role in STAT3 transcription ability. Mutation of STAT3 at lysine residue K685, a p300-acetylation site, inhibits dimerization of STAT3 (24, 25). In addition to K685, K49, and K87 at NTD of STAT3 can be acetylated by p300 in response to IL-6, which affects STAT3 downstream gene expression (26). Other than acetylation, K140 and K49 of STAT3 can be methylated and negatively and positively modulate STAT3-mediated transcription, respectively (27, 28). Furthermore, removal of sumoylation of STAT3 at K451 enhances its transcriptional activity by enhancing phosphorylation states (29). Together, these results suggest that multiple post-translational modifications of STAT3 modulate its activation and gene transcription.

Engagement of IFN-I to IFN receptor complex leads to activation of STAT1, STAT2, and STAT3 by tyrosine phosphorylation. STAT1 and STAT2 are considered to be the primordial signal mediators of IFN-I, as genetic ablation (30–33), hypomorphic mutation (33–35) or functional inactivation (35–38) of either molecule severely impairs the induction of IFN-stimulated genes (ISG) and IFN-I-mediated antiviral response in mice and humans. Nevertheless, STAT2 has also been reported to negatively regulate IFN-I response, either by constitutive phosphorylation at T387 to block ISGF3 formation and its DNA binding (39) or by IFN-I-induced phosphorylation at S287 (40) or S734 (41) with mechanisms yet to be defined. Moreover, STAT2 can serve as an adaptor to bridge the interaction between USP18 and IFNAR2, which inhibits ligand binding to the receptor, resulting in decreased receptor dimerization and signaling (42). These results suggest an emerging role of IFN-I signaling mediators in negative feedback regulation of IFN-I response.

While STAT3 is activated by IFN-I stimulation in various cell types in addition to STAT1 and STAT2 (43–47), the actual functions and biological significance of STAT3 in IFN-I response are less appreciated, probably due to relatively transient activation compared to STAT1 (3, 48, 49), impaired IFN-I-mediated, STAT3-dependent transcriptional activity (50, 51) or a dispensable role in some IFN-I-mediated activities (52–54). For example, IFN-I-induced growth stimulatory activity in the absence of either STAT1 or STAT2 is independent of STAT3 (52). STAT3 is non-essential and cannot compensate the loss of STAT1 for IFN-I- or IFN-II-induced antiviral response in U3A, a human epithelial cell line (53). Moreover, during Tfh differentiation, STAT3 is not involved in IFN-I-induced, STAT1-mediated upregulation of the key transcription factor Bcl6 in CD4 T cells (54). In fact, it has also been shown that low or no IFN-I-activated STAT3 is found in CD4 T cells of patients with relapsing remitting multiple sclerosis (55) and in normal melanocytes or melanoma cells from patients (56). However, it is still unclear why STAT3 is selectively activated by IFN-I in different cell types.

However, it becomes clear that STAT3 is not just activated by IFN-I, it also regulates IFN activity. In fact, emerging evidence suggests that STAT3 may function to fine-tune IFN-I response (57–60). Gain- and loss-of-function analyses suggest that STAT3 negatively regulates IFN-α-induced ISG expression and antiviral activity (60). STAT3KO Tfh cells displays a markedly elevated levels of a number of ISGs in addition to T-bet expression, resembling a Th1-like effector phenotype, which leads to impaired germinal center (GC) formation and antibody production during LCMV infection (61). Blockade of IFN-I signaling rescues the defect in LCMV-infected mice, suggesting a requirement of STAT3 for Tfh cell and a fine balance in signaling pathways following acute viral infection. Similarly, knockdown of STAT3 in highly STAT3 activated diffuse large B cell lymphoma (DLBCL) cells results in increased expression of several ISGs (58). Inhibition of STAT3 synergizes with lenalidomide, an IFN-I inducing agent, in suppressing the growth of DLBCL by augmenting IFN-I-induced cytotoxicity. In addition to ISGs, STAT3 deficiency also upregulates IFN-I production, which may contribute to enhanced antiviral response (60) and improved chemotherapeutic activity in tumors (62). Interestingly, STAT1 deficiency in murine macrophage results in a sustained activation of STAT3 in response to combined stimulation of TLR and IFN, leading to repressed production of cytokines like TNF and IL-12 (63). STAT3 depletion restores TLR-dependent inflammatory response in the absence of STAT1, suggesting a functional cross-regulation of STAT1 and STAT3 and an anti-inflammatory role of STAT3 in IFN and TLR response. Together, these studies suggest that STAT3 may function as one of the key regulatory molecules for IFN response.

Several potential mechanisms have been described to illustrate the direct and indirect actions of STAT3 to suppress IFN-I response. For example, STAT3 may sequester STAT1 and prevent it from forming functional homodimers, STAT3 can cooperate with repressors to inhibit ISGF3 binding to DNA, and STAT3 can directly reduce the expression of ISGF3 components. STAT3 can also induce a suppressor to attenuate IFN-I signaling or a miRNA to reduce the expression of ISGF3 components to indirectly block IFN-I response. These mechanisms will be elaborated further in the following section.

## Mechanisms of Negative Regulation of IFN-I by STAT3

### Sequestration of STAT1

**Figure 1:**
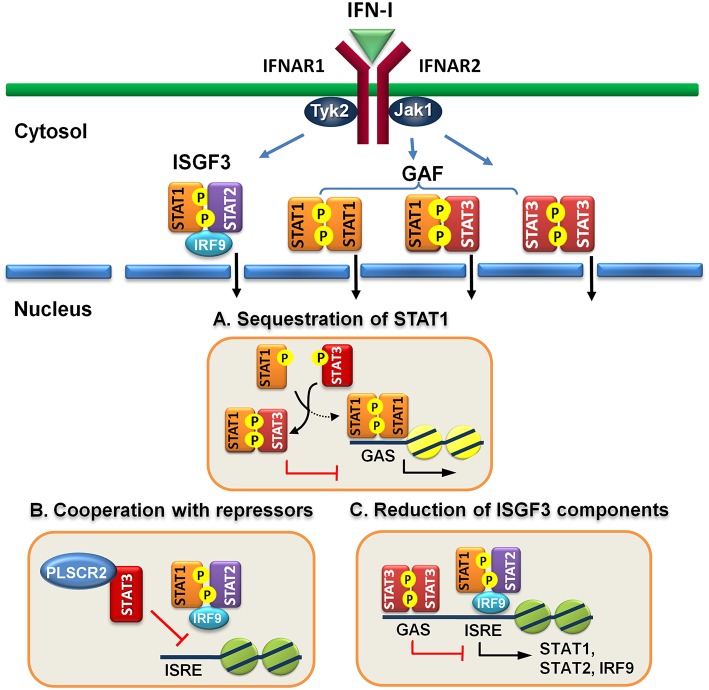
Direct regulatory mechanisms of STAT3 for IFN-I response. **(A)** Activated STAT3 sequesters activated STAT1 to form heterodimers and prevents STAT1 from forming functional homodimers to transactivate downstream genes. **(B)** STAT3 cooperates with other repressors, such as PLSCR2 to prevent ISGF3 from binding to DNA. **(C)** STAT3 binds directly to the promoters of ISGF3 components, including STAT1, STAT2, and IRF9 and suppress their expression.

Engagement of IFN-I to IFN receptor triggers the activation of STAT1, STAT2, and STAT3 and the formation of ISGF3 heterotrimer, consisting of STAT1, STAT2, and IRF9, and homodimers of STAT1:STAT1 or STAT3:STAT3 and heterodimer of STAT1:STAT3. It is conceivable that increased amounts of activated STAT3 can compete for binding with STAT1 to prevent the formation of STAT1:STAT1 homodimer. Indeed, overexpression of IFN-α-activated STAT3 inhibits STAT1-dependent gene expression, thereby downregulating the induction of proinflammatory cytokines, including CXCL9 and CXCL10 and a transcription factor IRF1, probably by sequestering activated STAT1 into STAT1:STAT3 heterodimers and reducing DNA binding of STAT1:STAT1 homodimers (57). Interestingly, increased amounts of activated STAT3 does not reduce ISGF3-driven expression of OAS and Mx2 genes. Instead, STAT3 seems to positively regulate their expression. Conversely, in mouse embryonic fibroblast cells lacking STAT3, IL-6 mediates IFNγ-like response with increased expression of MHC class II and antiviral state by inducing prolonged STAT1 activation and shifting homodimers of STAT1 or STAST3 and heterodimer of STAT1:STAT3 toward STAT1 homodimer only (64). Under physiological conditions, prolonged IFN-α treatment also affects STAT3-dependent IL-6 signaling by promoting formation of STAT1:STAT3 and STAT1:STAT1 complex, resulting in downregulation of STAT3 targets such as Bcl-X_L_, Mcl-1, and survivin and increased apoptosis (65). Likewise, IFN-α priming results in acquisition of pro-inflammatory function of IL-10, leading to expression of IFN-γ-inducible, STAT1-depedent genes (66). Therefore, IFN-I signaling can be antagonized by STAT3, at least, in two ways: to compete for STAT1 association and for DNA binding (Figure 1A).

### Cooperation With Repressors

STAT3 is reported to interact with many nuclear proteins to either activate or suppress the functions of STAT3 in transactivation ability and signaling pathways (67). For example, PDZ and LIM domain 2 (PDLIM2), a nuclear E3 ligase, which can interact with STAT3 and terminate its transcription by promoting degradation of STAT3 (68). HDAC1/2 forms a complex with SIN3 transcription regulator homolog A (Sin3a) to suppress the transcriptional activity of STAT3:STAT3 dimers through deacetylation (69, 70). In addition, DAXX directly interacts with STAT3 in the nucleus, leading to suppressing STAT3-mediated transactivation, probably through recruitment of a DNA methyltransferase (71, 72). STAT3 activation is therefore tightly regulated at multiple levels to prevent hyperactivation of STAT3-mediated pathologies, including cancers, autoimmune and inflammatory disorders (73).

However, these proteins are mainly limited to the control of the functions of STAT3 *per se*. Recently, we found that STAT3 interacts and cooperates with phospholipid scramblase 2 (PLSCR2) to negatively regulate IFN-I response (59). PLSCR2 is also an ISG and is predominantly located to the nucleus. PLSCR2 does not affect IFN-I-dependent phosphorylation of STAT1 and STAT2, nuclear translocation of activated STATs, or assembly of ISGF3 complex. Instead, PLSCR2 suppresses the recruitment of ISGF3 to ISRE of ISGs, in a STAT3-dependent manner, to fine-tune IFN-I response (Figure 1B). PLSCR2 deficiency results in increased ISG expression and antiviral activity. Mutations in palmitoylation motif of PLSCR2 impairs the interactions between PLSCR2 and STAT3, leading to blockade of the suppressive activity. Interestingly, expression profile analysis reveals that in addition to ISGs, genes involved in inflammatory response are also highly enriched in PLSCR2KO cells in response to IFN-I stimulation (59). This is consistent with enhanced inflammatory response upon TLR stimulation (74, 75) and in bowl diseases in the absence of STAT3 due to incapability of inducing SOCS3, a negative regulator of cytokine signaling (76–78) and the lack of signaling through the receptor of an anti-inflammatory cytokine such as IL-10 (79).

### Reduction of ISGF3 Components

Constitutive STAT3 activation by autocrine production of IL-6 and IL-10 is found in activated B cell-like (ABC) diffused large B cell lymphoma cell lines (DLBCL) (80, 81). Genome-wide analysis has identified ~2,200 STAT3 direct target genes which control different aspects of B cells, including activation, survival, proliferation, differentiation, and migration (58). STAT3 also regulates multiple oncogenic signaling pathways, including NF-κB, a cell-cycle checkpoint, PI3K/AKT/mTORC1, and STAT3 itself. Interestingly, constitutive STAT3 activation also suppresses the expression of ISGF3 components, such as IRF9, STAT1 and STAT2, and IRF7, a transcription factor for IFN-I production, through direct binding to the promoters of the genes (58). Therefore, STAT3 is able to directly reduce the levels of critical components in signaling pathway to weaken IFN-I response (Figure 1C).

**Figure 2:**
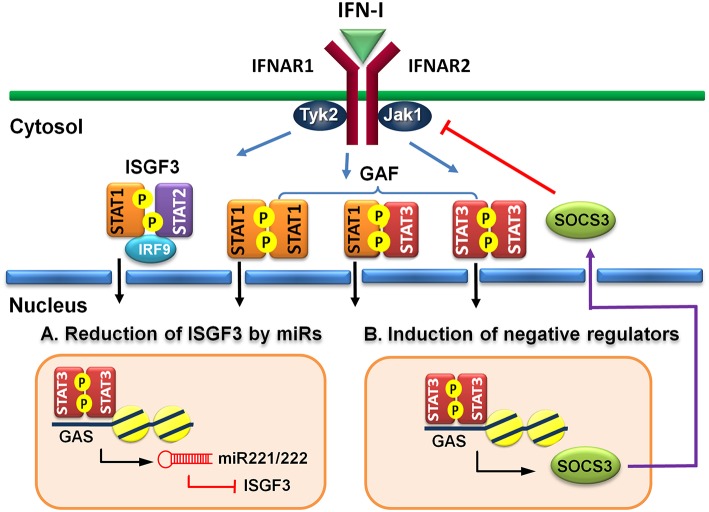
Indirect regulatory mechanisms of STAT3 for IFN-I responses. **(A)** STAT3 induces microRNA miR-221/222 to target ISGF3 components, including STAT1, STAT2, and IRF9 to reduce their *de novo* protein synthesis. **(B)** STAT3 induces a negative regulator, such as SOCS3 to block IFN-I signaling.

MicroRNAs (miRs) are known to regulate IFN signaling pathways (82, 83). IFN-γ-activated STAT1 binds directly to the promoter of miR155 and induces its expression (84). Moreover, virus infection-induced miR155 also targets SOCS1 to feedback promote IFN-I-mediated antiviral activity (85). Therefore, STAT1 activation positively regulates its function by up-regulating miR-155 and down-regulating a STAT inhibitory factor SOCS1. STAT3, on other hand, stimulates miR-221/222 expression, which in turn targets PDLIM2 to stabilize and increase STAT3 levels (86). Knockdown of miR-221/222 results in upregulation of members of the IFN-α signaling pathway, including STAT1, STAT2, IRF9, and several ISGs in a human glioma cell line (87). Virus infection-induced upregulation of miR221 in peritoneal macrophages negatively regulates innate antiviral response against VSV (88). Therefore, STAT3-dependent transcription of miR-221/222 may function to downregulate STAT1 and STAT2 expression indirectly to antagonize IFN-I response (Figure 2A).

### Induction of Negative Regulators

Suppressors of cytokine signaling (SOCS) family proteins are ISGs that feedback regulate cytokine signaling through various ways, including blocking receptor docking by activated STATs, inhibiting JAK kinase activity, and promoting degradation of activated JAKs and receptors (89, 90). IFN-I induces SOCS1 and SOCS3 expression in a STAT1- and STAT3-dependent manner, respectively (91). In addition to feedback regulation, the signaling pathways of STAT1 and STAT3 can cross-regulate by the induced SOCS1 and SOCS3 reciprocally (92, 93). Constitutive expression of SOCS3 inhibits IFN-α-induced STAT1 phosphorylation, ISG expression and anti-proliferative activity (94). HSV-1 (95) or IAV (96) infection-induced SOCS3 is responsible for the suppression of signaling and production of IFN-I and impaired antiviral response. Moreover, hepatic SOCS3 expression is also strongly associated with non-responsiveness to IFN-I therapy in HCV patients (97). Therefore, STAT3 can indirectly cross-regulate STAT1-mediated signaling and ISG expression at multiple layers of feedback control through induced SOCS3 (Figure 2B).

## Viral Strategies to Exploit STAT3

Given that STAT3 can exert negative effects on IFN-I response, it is conceivable that viruses may exploit STAT3 to evade IFN-I-mediated antiviral immunity to facilitate their replication. Indeed, porcine epidemic diarrhea virus is shown to trigger STAT3 activation via stimulated EGFR signaling to enhance virus replication in an intestinal epithelial cell line (98). Inhibitors or siRNA to EGFR result in augmented expression of IFN-I and ISG genes and decreased viral yield. Similar results are also observed using the same approaches to block STAT3 activation, suggesting that attenuation of antiviral activity by EGFR activation requires STAT3 signaling pathway. EGFR- and IFN-signaling crosstalk is also known to play a role in regulating HCV replication (99). Erlotinib, an EGFR inhibitor, and IFN-α synergize to inhibit HCV infection in a hepatoma cell line (100). While STAT3 silencing or inhibition suppresses HCV infection, SOCS silencing impairs the synergistic antiviral activity of IFN-α and erlotinib. Therefore, EGFR may impair IFN antiviral response by suppressing SOCS3 expression, which relieves SOCS3-mediated antagonism of STAT3, thereby promoting virus replication (100).

Although virus targeting and inhibiting STAT3 seems to be counterintuitive because of its negative role in IFN-I response (57–60), several viruses are reported to degrade STAT3 protein or suppress its functions. For example, the V protein of Mumps virus (MuV) catalyzes proteasomal degradation of STAT1 and STAT3, resulting in blockade of IFN-I, IFN-II, and prevention of the responses to interleukin-6 and v-Src signals and induction of apoptosis in STAT3-dependent multiple myeloma cells and transformed murine fibroblasts (101). Hepatitis E virus (HEV) viral ORF3 protein (pORF3) blocks the nuclear translocation of p-STAT3, probably by impeding endocytosis of EGFR, resulting in downregulation of STAT3-stimulated acute-phase gene-driven reporter activity (102). While, the absence of STAT3 during MuV infection may reduce pro-inflammatory activity of IL-6 and IFN-γ, functional blockade of STAT3 by HEV may result in downregulation of the acute-phase response, a major determinant of inflammation in the host.

In fact, several viruses also boost or attenuate STAT3 functions to perturb immune response, alter cell architecture and tissue organization, prevent apoptosis or trigger cellular transformation to facilitate their replication, which have been thoroughly reviewed elsewhere (103, 104) and will not be discussed further.

## Evolutionarily Conserved Feedback Regulation by STAT

The cytokine receptor (CytoR)-JAK-STAT is a highly conserved signaling pathway, which expands extensively in bilateria during early vertebrate evolution and is concurrent with the development of adaptive immune system (105, 106). In early jawed vertebrates, the regulation of IFN has been established through two rounds of whole-genome duplication that occurs between invertebrates and vertebrates to provide expanded signaling molecules, such as positive regulators, JAKs, STATs and IRFs, and negative regulators, protein inhibitor of activated STAT (PIAS) and SOCS (107).

There is only one STAT protein in *Drosophila* that is stat92E which is required for normal development of several tissues, including embryonic segmentation, imaginal discs, blood cells, and germ cells (108). A crustacean Pm-STAT is also identified from giant tiger prawn (*Penaeus monodon*) and the phosphorylated form of Pm-STAT is increased in lymphoid organ of the shrimp following white spot syndrome virus (WSSV) infection (109). RNA silencing of Lv-STAT in whiteleg shrimp (*Litopenaeus vannamei*) significantly reduces the copy number of WSSV and the mortality caused by WSSV infection (110). Moreover, treatment of a specific inhibitor of STAT3 (S3I-201) in hematopoietic tissue of crayfish (*Cherax quadricarinatus*) also decreases WSSV titers, suggesting a proviral role of the invertebrate STAT protein (110).

In addition to shrimp, an Ec-STAT3 identified from orange-spotted grouper (*Epinephelus coioides*) is activated and induced to translocate into nucleus following Singapore grouper iridovirus (SGIV) infection. Inhibition of Ec-STAT3 by RNA silencing or a small molecule inhibitor decreases SGIV replication and induces cell cycle arrest and downregulates the expression of prosurvival genes, such as Bcl-2, Bcl-x_L_, and Bax. Ec-STAT3 is also activated in fish by red-spotted grouper nervous necrosis virus (RGNNV). While inhibition of Ec-STAT3 does not affect RGNNV replication, virus infection-induced vacuolation and autophagy are significantly increased (111). Moreover, the expression of several proinflammatory factors, including TNFα, IL-1β, and IL-8, is also mediated by Ec-STAT3 during infection.

Therefore, these results suggest that microbial infection-triggered STAT signaling pathway is well conserved from invertebrates to vertebrates and that proviral and prosurvial role of STAT3 are probably also preserved from shrimp, fish to mammals, although the feedback regulation of IFN-I response by STAT3 in shrimp and fish remains unclear.

## Is STAT3 Druggable or Undruggable?

As an oncogene and is activated in a wide range of malignant cells, STAT3 is considered to be one of the most important therapeutic targets, particularly, for cancers (112). However, there are still lack of clinically available STAT3 inhibitors due to insufficient potency and/or selectivity (113, 114). Currently, the STAT3 inhibitors under development include three categories: small molecule compounds, peptide-based molecules, and double-stranded oligodeoxynucleotide decoy (ODN-decoy) (115). Usually, small molecule compounds and peptide-based inhibitors are designed in a way to effectively reduce tyrosine phosphorylation and/or dimerization of STAT3 and block STAT3-mediated transcriptional activity. For example, SH2 domain of STAT3 is the main target of STAT3 inhibitors which are known to occupy or dock to pY-Tyr705-binding pocket. However, it is also highly homologous to SH2 domain of STAT1. Likewise, the DBD of STAT3 is also highly similar to that of STAT1. Therefore, the chances of cross-reacting and simultaneous suppression of the activity of STAT1 and STAT3 by an inhibitor are high. Indeed, inhibitors like Stattic and S3I-201 that target STAT3 SH2 domain are also reported to inhibit STAT1 phosphorylation and nuclear translocation (116, 117).

Since both STAT3 and STAT1 can recognize the same GAS element of some genes, it comes as no surprise that STAT3 DBD-targeting decoy ODN also binds STAT1 and reduces STAT1-dependent IFNγ-induced cell death (118). Therefore, the inhibitors targeting to either SH2 or DBD domain of STAT3 are likely to suppress STAT1-mediated signaling. In addition, targeting upstream of STAT3, such as JAKs, is also likely to exert a board spectrum of inhibitory effect on different STAT proteins. For example, AG490, AZD1480, and Sorafenib are also able to potently suppress STAT1 phosphorylation in addition to STAT3 phosphorylation (119–121).

When STAT3 inhibitors are used to boost antiviral responses, it is anticipated that STAT1-mediated antiviral responses will also be attenuated due to cross-reactivity. Nevertheless, several STAT3 inhibitors have been shown to enhance antiviral response *in vitro* or *in vivo* and display some therapeutic potential. For example, pretreatment of S3I-201 can reduce viral replication in human cytomegalovirus (HCMV), varicella-zoster virus, SGIV, or vesicular stomatitis virus (122–126). Moreover, Stattic can also reduce HCMV replication (126). Although these studies usually attribute the reduced viral replication to the proviral role of STAT3, it is also likely that reduced SOCS3 expression derepresses STAT1 to contribute to antiviral responses.

Despite the concerns raised in many literatures about the potential problems in developing STAT3 inhibitors, some of them are already in clinical trials, including 3 inhibitors targeting to SH2 of STAT3 and 1 inhibitor as an antisense ODN (127). Therefore, there is no definitely answer yet as STAT3 is druggable or not. Nevertheless, different approaches may need to be implanted to provide viable solutions for developing drugs that can selectively block the activity of STAT3.

## Conclusions and Perspectives

While accumulating evidence suggests that STAT3 is a negative regulator of the IFN-I response, it only represents one of many other regulatory mechanisms for IFN-I signaling (128), suggesting the importance of balancing the pluripotent activities of IFN-I. In fact, this notion becomes evident with the emerging type I interferonopathies of autoinflammatory diseases, which are Mendelian disorders associated with an up-regulation of IFN-I signaling as a novel type of human inborn errors of immunity, including Aicardi-Goutières syndromes (AGS), familial chilblain lupus (FCL), bilateral striatal necrosis (BSN), STING-associated vasculopathy with onset in infancy (SAVI)…etc. (129). Mutations in STAT3 is known to cause many diseases in humans, including loss-of-function-induced autosomal dominant hyper IgE syndrome (AD-HIES), gain-of-function-induced malignancies and autoimmunity (73). However, it remains to be determined if functional deficiency of STAT3 will lead to type I interferonopathies.

Targeting STAT3 as a therapeutic approach for cancers and other diseases must take the multifaceted functions of STAT3 into consideration. As shown in this review that STAT3 not just possesses proviral and prosurvival activities, it also exhibits seemingly paradoxical roles in inflammation (130) and immune modulation (5, 131, 132). Moreover, due to sequence conservation between STAT1 and STAT3, developing highly selective drugs for STAT3 without affecting STAT1 is desirable, which is also the challenge for precision medicine to deal with these diseases.

## Author Contributions

All authors listed have made a substantial, direct and intellectual contribution to the work, and approved it for publication.

### Conflict of Interest Statement

The authors declare that the research was conducted in the absence of any commercial or financial relationships that could be construed as a potential conflict of interest.
